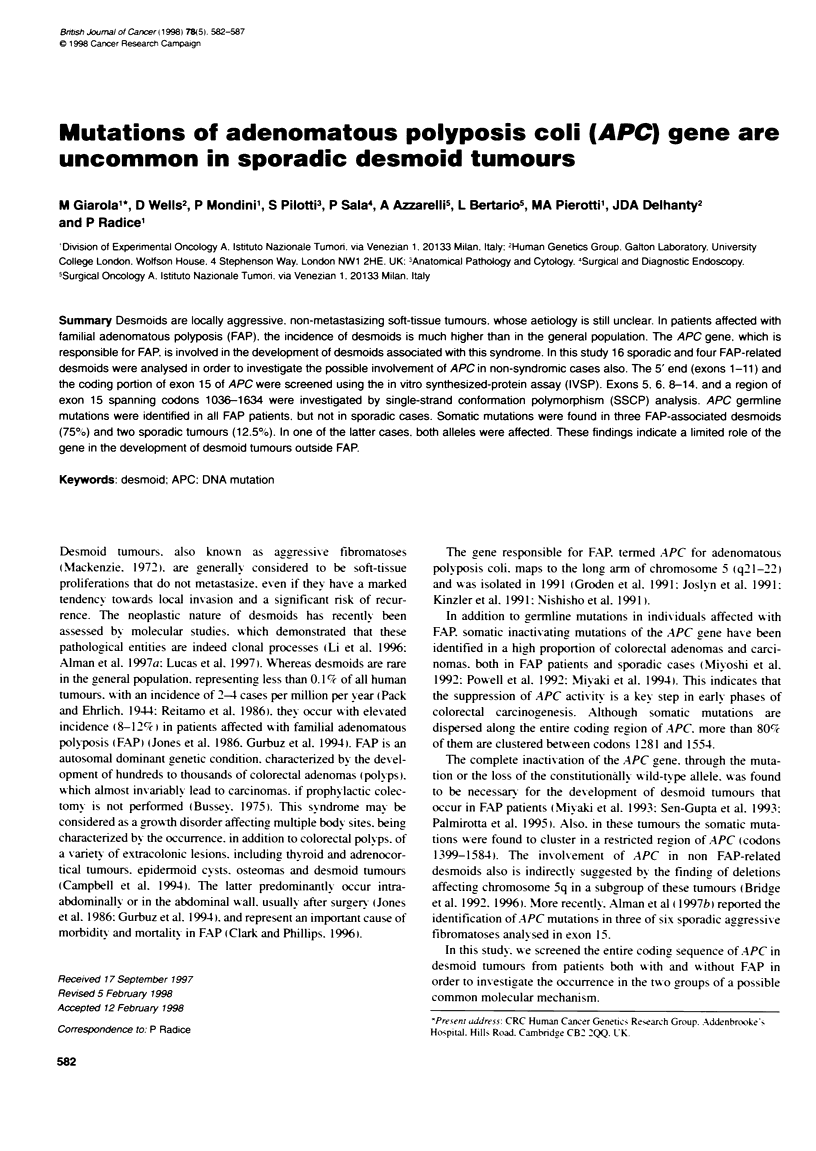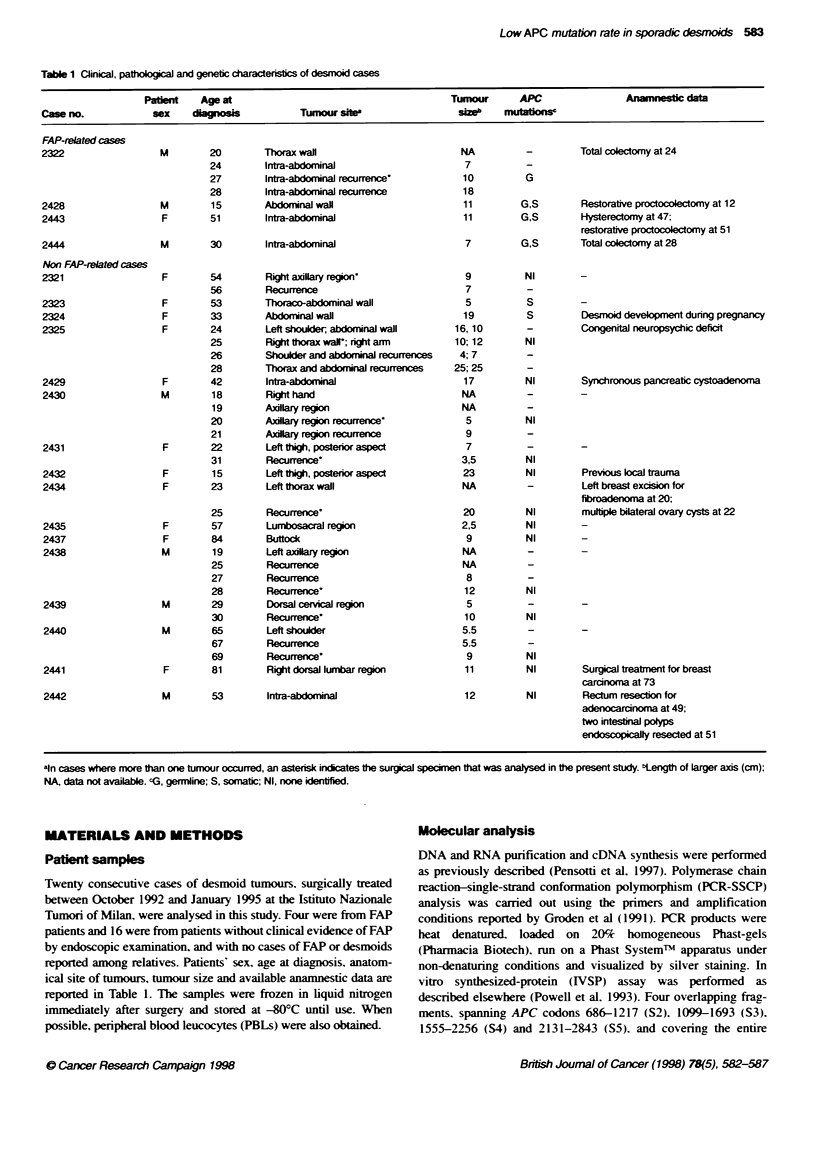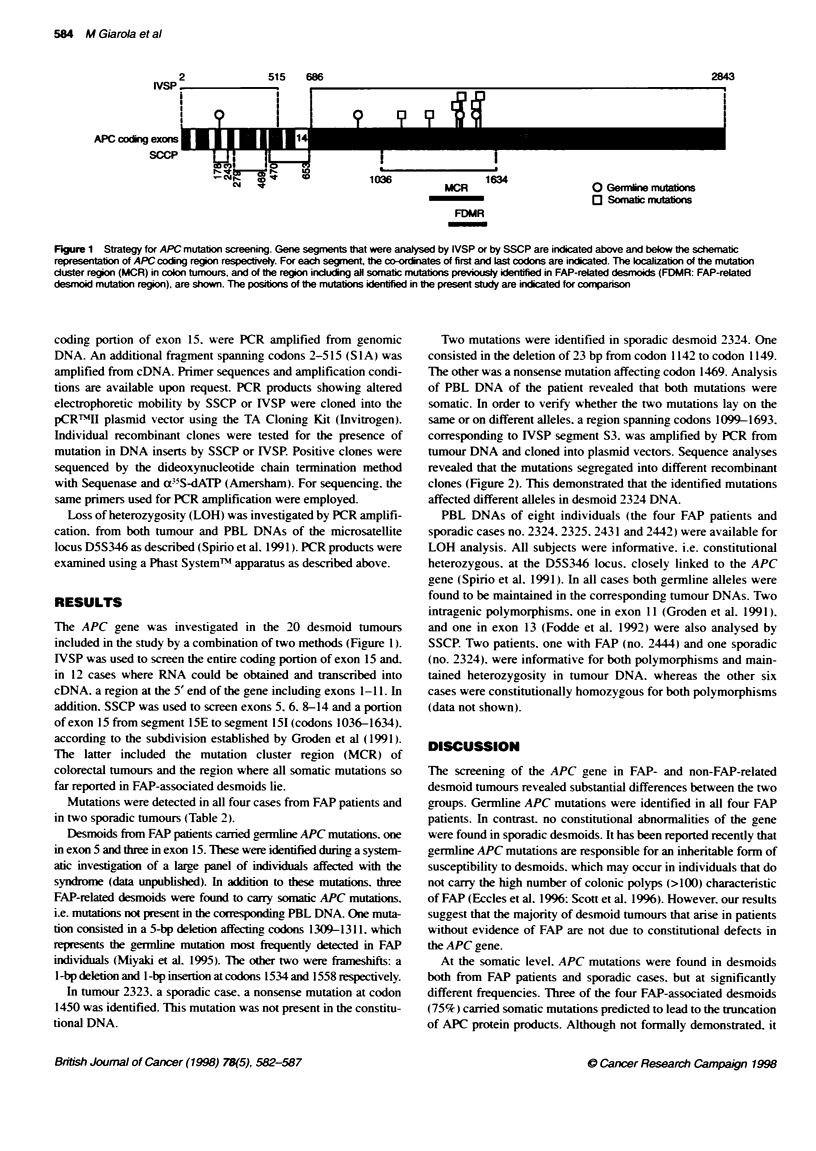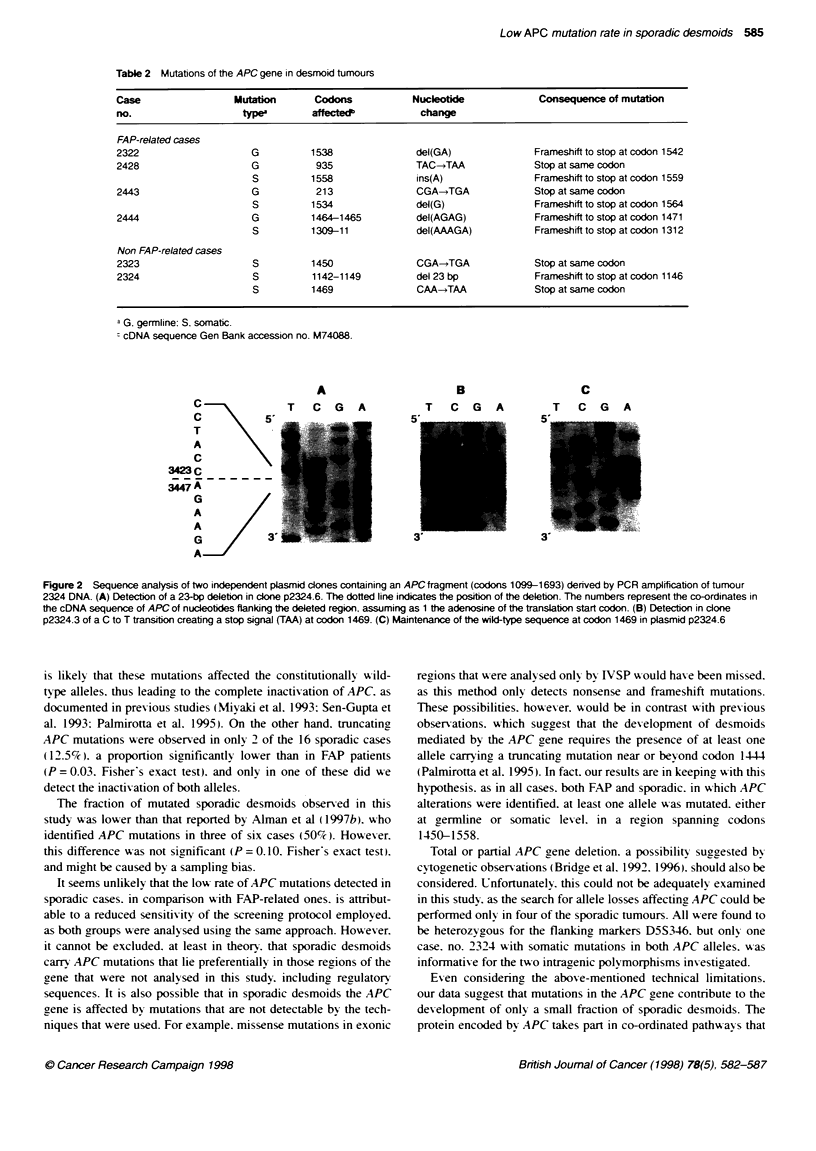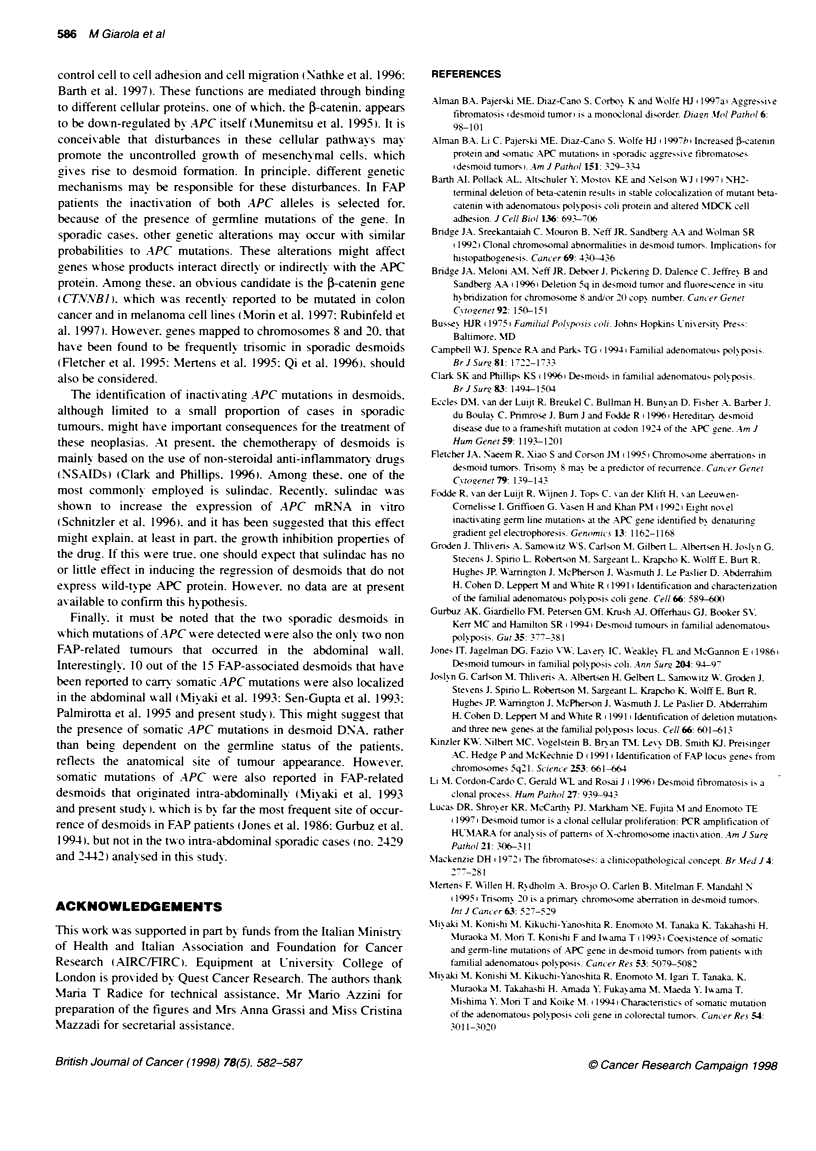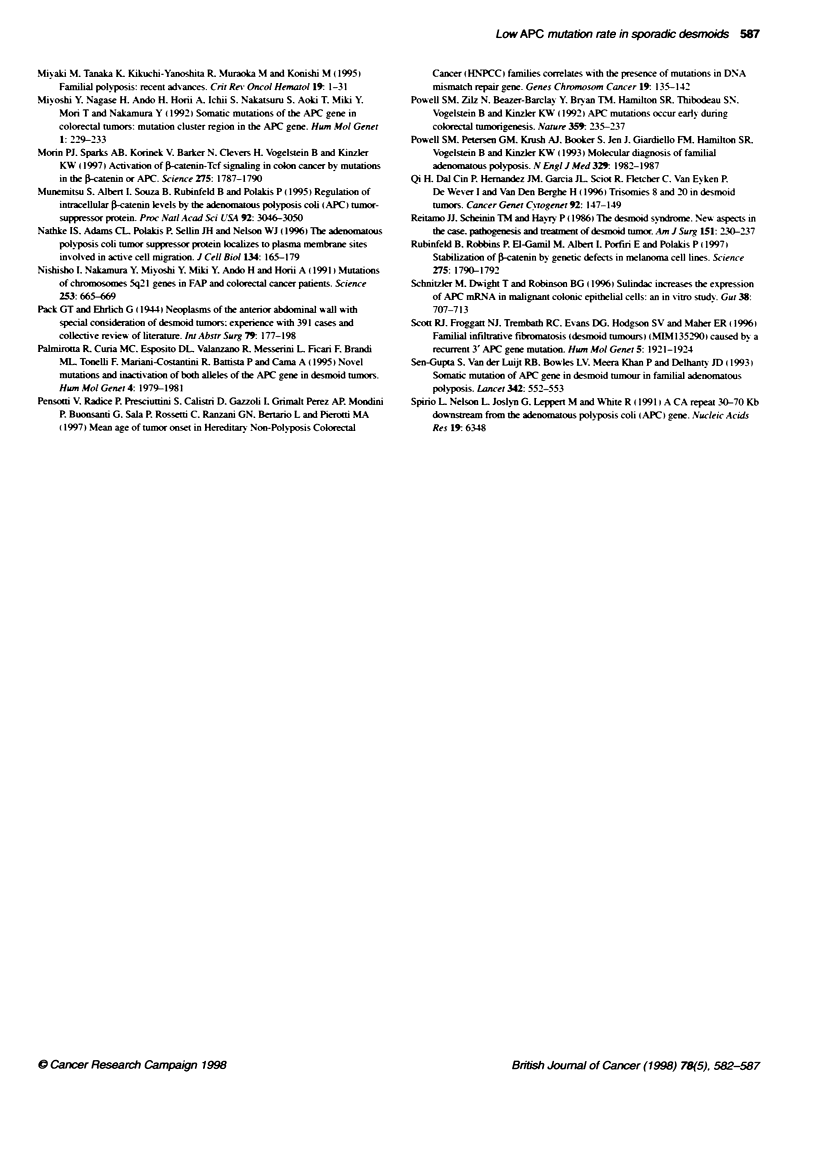# Mutations of adenomatous polyposis coli (APC) gene are uncommon in sporadic desmoid tumours.

**DOI:** 10.1038/bjc.1998.544

**Published:** 1998-09

**Authors:** M. Giarola, D. Wells, P. Mondini, S. Pilotti, P. Sala, A. Azzarelli, L. Bertario, M. A. Pierotti, J. D. Delhanty, P. Radice

**Affiliations:** Division of Experimental Oncology A, Instituto Nazionale Tumori, Milan, Italy.

## Abstract

**Images:**


British Journal of Cancer (1998) 78(5). 582–587
© 1998 Cancer Research Campaign

### Mutations of adenomatous polyposis coli (*APC*) gene are uncommon in sporadic desmoid tumours

**M Giarola[1]*, D Wells[2], P Mondini[1], S Pilotti[3], P Sala[4], A Azzarelli[5], L Bertario[5], MA Pierotti[1], JDA Delhanty[2] and P Radice[1]**

[1]Division of Experimental Oncology A. Istituto Nazionale Tumori. via Venezian 1. 20133 Milan. Italy: [2]Human Genetics Group. Galton Laboratory. University College London. Wolfson House. 4 Stephenson Way. London NW1 2HE. UK: [3]Anatomical Pathology and Cytology. [4]Surgical and Diagnostic Endoscopy. [5]Surgical Oncology A. Istituto Nazionale Tumori. via Venezian 1. 20133 Milan. Italy

**Summary** Desmoids are locally aggressive. non-metastasizing soft-tissue tumours. whose aetiology is still unclear. In patients affected with familial adenomatous polyposis (FAP). the incidence of desmoids is much higher than in the general population. The *APC* gene. which is responsible for FAP. is involved in the development of desmoids associated with this syndrome. In this study 16 sporadic and four FAP-related desmoids were analysed in order to investigate the possible involvement of *APC* in non-syndromic cases also. The 5′ end (exons 1–11) and the coding portion of exon 15 of *APC* were screened using the in vitro synthesized-protein assay (IVSP). Exons 5. 6. 8–14. and a region of exon 15 spanning codons 1036–1634 were investigated by single-strand conformation polymorphism (SSCP) analysis. *APC* germline mutations were identified in all FAP patients. but not in sporadic cases. Somatic mutations were found in three FAP-associated desmoids (75%) and two sporadic tumours (12.5%). In one of the latter cases. both alleles were affected. These findings indicate a limited role of the gene in the development of desmoid tumours outside FAP.

**Keywords**: desmoid; APC; DNA mutation

Desmoid tumours. also known as aggressive fibromatoses (Mackenzie. 1972). are generally considered to be soft-tissue proliferations that do not metastasize. even if they have a marked tendency towards local invasion and a significant risk of recurrence. The neoplastic nature of desmoids has recently been assessed by molecular studies. which demonstrated that these pathological entities are indeed clonal processes (Li et al. 1996: Alman et al. 1997*a*: Lucas et al. 1997). Whereas desmoids are rare in the general population. representing less than 0.1% of all human tumours. with an incidence of 2–4 cases per million per year (Pack and Ehrlich. 1944: Reitamo et al. 1986). they occur with elevated incidence (8–12%) in patients affected with familial adenomatous polyposis (FAP) (Jones et al. 1986. Gurbuz et al. 1994). FAP is an autosomal dominant genetic condition. characterized by the development of hundreds to thousands of colorectal adenomas (polyps). which almost invariably lead to carcinomas. if prophylactic colectomy is not performed (Bussey. 1975). This syndrome may be considered as a growth disorder affecting multiple body sites. being characterized by the occurrence. in addition to colorectal polyps. of a variety of extracolonic lesions. including thyroid and adrenocortical tumours. epidermoid cysts. osteomas and desmoid tumours (Campbell et al. 1994). The latter predominantly occur intra-abdominally or in the abdominal wall. usually after surgery (Jones et al. 1986: Gurbuz et al. 1994). and represent an important cause of morbidity and mortality in FAP (Clark and Phillips. 1996).

*Received 17 September 1997*
*Revised 5 February 1998*
*Accepted 12 February 1998*

*Correspondence to:* P Radice

The gene responsible for FAP. termed *APC* for adenomatous polyposis coli. maps to the long arm of chromosome 5 (q21–22) and was isolated in 1991 (Groden et al. 1991: Joslyn et al. 1991: Kinzler et al. 1991: Nishisho et al. 1991).

In addition to germline mutations in individuals affected with FAP. somatic inactivating mutations of the *APC* gene have been identified in a high proportion of colorectal adenomas and carcinomas. both in FAP patients and sporadic cases (Miyoshi et al. 1992: Powell et al. 1992: Miyaki et al. 1994). This indicates that the suppression of *APC* activity is a key step in early phases of colorectal carcinogenesis. Although somatic mutations are dispersed along the entire coding region of *APC*. more than 80% of them are clustered between codons 1281 and 1554.

The complete inactivation of the *APC* gene. through the mutation or the loss of the constitutionally wild-type allele. was found to be necessary for the development of desmoid tumours that occur in FAP patients (Miyaki et al. 1993: Sen-Gupta et al. 1993: Palmirotta et al. 1995). Also. in these tumours the somatic mutations were found to cluster in a restricted region of *APC* (codons 1399–1584). The involvement of *APC* in non FAP-related desmoids also is indirectly suggested by the finding of deletions affecting chromosome 5q in a subgroup of these tumours (Bridge et al. 1992. 1996). More recently. Alman et al (1997*b*) reported the identification of *APC* mutations in three of six sporadic aggressive fibromatoses analysed in exon 15.

In this study. we screened the entire coding sequence of *APC* in desmoid tumours from patients both with and without FAP in order to investigate the occurrence in the two groups of a possible common molecular mechanism.

**Present address*: CRC Human Cancer Genetics Research Group. Addenbrooke's Hospital. Hills Road. Cambridge CB2 2QQ. UK.

**Table 1** Clinical, pathological and genetic characteristics of desmoid cases

| Case no. | Patient sex | Age at diagnosis | Tumour site[a] | Tumour size[b] | *APC* mutations[c] | Anamnestic data |
|---|---|---|---|---|---|---|
| *FAP-related cases* | | | | | | |
| 2322 | M | 20 | Thorax wall | NA | – | Total colectomy at 24 |
| | | 24 | Intra-abdominal | 7 | – | |
| | | 27 | Intra-abdominal recurrence* | 10 | G | |
| | | 28 | Intra-abdominal recurrence | 18 | | |
| 2428 | M | 15 | Abdominal wall | 11 | G,S | Restorative proctocolectomy at 12 |
| 2443 | F | 51 | Intra-abdominal | 11 | G,S | Hysterectomy at 47; restorative proctocolectomy at 51 |
| 2444 | M | 30 | Intra-abdominal | 7 | G,S | Total colectomy at 28 |
| *Non FAP-related cases* | | | | | | |
| 2321 | F | 54 | Right axillary region* | 9 | NI | – |
| | | 56 | Recurrence | 7 | – | |
| 2323 | F | 53 | Thoraco-abdominal wall | 5 | S | – |
| 2324 | F | 33 | Abdominal wall | 19 | S | Desmoid development during pregnancy |
| 2325 | F | 24 | Left shoulder; abdominal wall | 16, 10 | – | Congenital neuropsychic deficit |
| | | 25 | Right thorax wall*; right arm | 10; 12 | NI | |
| | | 26 | Shoulder and abdominal recurrences | 4; 7 | – | |
| | | 28 | Thorax and abdominal recurrences | 25; 25 | – | |
| 2429 | F | 42 | Intra-abdominal | 17 | NI | Synchronous pancreatic cystoadenoma |
| 2430 | M | 18 | Right hand | NA | – | – |
| | | 19 | Axillary region | NA | – | |
| | | 20 | Axillary region recurrence* | 5 | NI | |
| | | 21 | Axillary region recurrence | 9 | – | |
| 2431 | F | 22 | Left thigh, posterior aspect | 7 | – | – |
| | | 31 | Recurrence* | 3,5 | NI | |
| 2432 | F | 15 | Left thigh, posterior aspect | 23 | NI | Previous local trauma |
| 2434 | F | 23 | Left thorax wall | NA | – | Left breast excision for fibroadenoma at 20; |
| | | 25 | Recurrence* | 20 | NI | multiple bilateral ovary cysts at 22 |
| 2435 | F | 57 | Lumbosacral region | 2,5 | NI | – |
| 2437 | F | 84 | Buttock | 9 | NI | – |
| 2438 | M | 19 | Left axillary region | NA | – | – |
| | | 25 | Recurrence | NA | – | |
| | | 27 | Recurrence | 8 | – | |
| | | 28 | Recurrence* | 12 | NI | |
| 2439 | M | 29 | Dorsal cervical region | 5 | – | – |
| | | 30 | Recurrence* | 10 | NI | |
| 2440 | M | 65 | Left shoulder | 5.5 | – | – |
| | | 67 | Recurrence | 5.5 | – | |
| | | 69 | Recurrence* | 9 | NI | |
| 2441 | F | 81 | Right dorsal lumbar region | 11 | NI | Surgical treatment for breast carcinoma at 73 |
| 2442 | M | 53 | Intra-abdominal | 12 | NI | Rectum resection for adenocarcinoma at 49; two intestinal polyps endoscopically resected at 51 |

[a]In cases where more than one tumour occurred, an asterisk indicates the surgical specimen that was analysed in the present study. [b]Length of larger axis (cm); NA, data not available. [c]G, germline; S, somatic; NI, none identified.

### MATERIALS AND METHODS

### Patient samples

Twenty consecutive cases of desmoid tumours, surgically treated between October 1992 and January 1995 at the Istituto Nazionale Tumori of Milan, were analysed in this study. Four were from FAP patients and 16 were from patients without clinical evidence of FAP by endoscopic examination, and with no cases of FAP or desmoids reported among relatives. Patients' sex, age at diagnosis, anatomical site of tumours, tumour size and available anamnestic data are reported in Table 1. The samples were frozen in liquid nitrogen immediately after surgery and stored at –80°C until use. When possible, peripheral blood leucocytes (PBLs) were also obtained.

### Molecular analysis

DNA and RNA purification and cDNA synthesis were performed as previously described (Pensotti et al, 1997). Polymerase chain reaction–single-strand conformation polymorphism (PCR-SSCP) analysis was carried out using the primers and amplification conditions reported by Groden et al (1991). PCR products were heat denatured, loaded on 20% homogeneous Phast-gels (Pharmacia Biotech), run on a Phast System™ apparatus under non-denaturing conditions and visualized by silver staining. In vitro synthesized-protein (IVSP) assay was performed as described elsewhere (Powell et al, 1993). Four overlapping fragments, spanning *APC* codons 686–1217 (S2), 1099–1693 (S3), 1555–2256 (S4) and 2131–2843 (S5), and covering the entire

© Cancer Research Campaign 1998 

**Figure 1** Strategy for *APC* mutation screening. Gene segments that were analysed by IVSP or by SSCP are indicated above and below the schematic representation of *APC* coding region respectively. For each segment, the co-ordinates of first and last codons are indicated. The localization of the mutation cluster region (MCR) in colon tumours, and of the region including all somatic mutations previously identified in FAP-related desmoids (FDMR: FAP-related desmoid mutation region), are shown. The positions of the mutations identified in the present study are indicated for comparison coding portion of exon 15, were PCR amplified from genomic DNA. An additional fragment spanning codons 2–515 (S1A) was amplified from cDNA. Primer sequences and amplification conditions are available upon request. PCR products showing altered electrophoretic mobility by SSCP or IVSP were cloned into the pCR™II plasmid vector using the TA Cloning Kit (Invitrogen). Individual recombinant clones were tested for the presence of mutation in DNA inserts by SSCP or IVSP. Positive clones were sequenced by the dideoxynucleotide chain termination method with Sequenase and $\alpha^{35}S$-dATP (Amersham). For sequencing, the same primers used for PCR amplification were employed.

Loss of heterozygosity (LOH) was investigated by PCR amplification, from both tumour and PBL DNAs of the microsatellite locus D5S346 as described (Spirio et al, 1991). PCR products were examined using a Phast System™ apparatus as described above.

### RESULTS

The *APC* gene was investigated in the 20 desmoid tumours included in the study by a combination of two methods (Figure 1). IVSP was used to screen the entire coding portion of exon 15 and, in 12 cases where RNA could be obtained and transcribed into cDNA, a region at the 5′ end of the gene including exons 1–11. In addition, SSCP was used to screen exons 5, 6, 8–14 and a portion of exon 15 from segment 15E to segment 15I (codons 1036–1634), according to the subdivision established by Groden et al (1991). The latter included the mutation cluster region (MCR) of colorectal tumours and the region where all somatic mutations so far reported in FAP-associated desmoids lie.

Mutations were detected in all four cases from FAP patients and in two sporadic tumours (Table 2).

Desmoids from FAP patients carried germline *APC* mutations, one in exon 5 and three in exon 15. These were identified during a systematic investigation of a large panel of individuals affected with the syndrome (data unpublished). In addition to these mutations, three FAP-related desmoids were found to carry somatic *APC* mutations, i.e. mutations not present in the corresponding PBL DNA. One mutation consisted in a 5-bp deletion affecting codons 1309–1311, which represents the germline mutation most frequently detected in FAP individuals (Miyaki et al, 1995). The other two were frameshifts: a 1-bp deletion and 1-bp insertion at codons 1534 and 1558 respectively.

In tumour 2323, a sporadic case, a nonsense mutation at codon 1450 was identified. This mutation was not present in the constitutional DNA.

Two mutations were identified in sporadic desmoid 2324. One consisted in the deletion of 23 bp from codon 1142 to codon 1149. The other was a nonsense mutation affecting codon 1469. Analysis of PBL DNA of the patient revealed that both mutations were somatic. In order to verify whether the two mutations lay on the same or on different alleles, a region spanning codons 1099–1693, corresponding to IVSP segment S3, was amplified by PCR from tumour DNA and cloned into plasmid vectors. Sequence analyses revealed that the mutations segregated into different recombinant clones (Figure 2). This demonstrated that the identified mutations affected different alleles in desmoid 2324 DNA.

PBL DNAs of eight individuals (the four FAP patients and sporadic cases no. 2324, 2325, 2431 and 2442) were available for LOH analysis. All subjects were informative, i.e. constitutional heterozygous, at the D5S346 locus, closely linked to the *APC* gene (Spirio et al, 1991). In all cases both germline alleles were found to be maintained in the corresponding tumour DNAs. Two intragenic polymorphisms, one in exon 11 (Groden et al, 1991), and one in exon 13 (Fodde et al, 1992) were also analysed by SSCP. Two patients, one with FAP (no. 2444) and one sporadic (no. 2324), were informative for both polymorphisms and maintained heterozygosity in tumour DNA, whereas the other six cases were constitutionally homozygous for both polymorphisms (data not shown).

### DISCUSSION

The screening of the *APC* gene in FAP- and non-FAP-related desmoid tumours revealed substantial differences between the two groups. Germline *APC* mutations were identified in all four FAP patients. In contrast, no constitutional abnormalities of the gene were found in sporadic desmoids. It has been reported recently that germline *APC* mutations are responsible for an inheritable form of susceptibility to desmoids, which may occur in individuals that do not carry the high number of colonic polyps (>100) characteristic of FAP (Eccles et al, 1996; Scott et al, 1996). However, our results suggest that the majority of desmoid tumours that arise in patients without evidence of FAP are not due to constitutional defects in the *APC* gene.

At the somatic level, *APC* mutations were found in desmoids both from FAP patients and sporadic cases, but at significantly different frequencies. Three of the four FAP-associated desmoids (75%) carried somatic mutations predicted to lead to the truncation of APC protein products. Although not formally demonstrated, it

© Cancer Research Campaign 1998

**Table 2** Mutations of the *APC* gene in desmoid tumours

| Case no. | Mutation type[a] | Codons affected[b] | Nucleotide change | Consequence of mutation |
|---|---|---|---|---|
| *FAP-related cases* | | | | |
| 2322 | G | 1538 | del(GA) | Frameshift to stop at codon 1542 |
| 2428 | G | 935 | TAC→TAA | Stop at same codon |
| | S | 1558 | ins(A) | Frameshift to stop at codon 1559 |
| 2443 | G | 213 | CGA→TGA | Stop at same codon |
| | S | 1534 | del(G) | Frameshift to stop at codon 1564 |
| 2444 | G | 1464–1465 | del(AGAG) | Frameshift to stop at codon 1471 |
| | S | 1309–11 | del(AAAGA) | Frameshift to stop at codon 1312 |
| *Non FAP-related cases* | | | | |
| 2323 | S | 1450 | CGA→TGA | Stop at same codon |
| 2324 | S | 1142–1149 | del 23 bp | Frameshift to stop at codon 1146 |
| | S | 1469 | CAA→TAA | Stop at same codon |

[a] G. germline: S. somatic.
[b] cDNA sequence Gen Bank accession no. M74088.

**Figure 2** Sequence analysis of two independent plasmid clones containing an *APC* fragment (codons 1099–1693) derived by PCR amplification of tumour 2324 DNA. (**A**) Detection of a 23-bp deletion in clone p2324.6. The dotted line indicates the position of the deletion. The numbers represent the co-ordinates in the cDNA sequence of *APC* of nucleotides flanking the deleted region, assuming as 1 the adenosine of the translation start codon. (**B**) Detection in clone p2324.3 of a C to T transition creating a stop signal (TAA) at codon 1469. (**C**) Maintenance of the wild-type sequence at codon 1469 in plasmid p2324.6

is likely that these mutations affected the constitutionally wild-type alleles, thus leading to the complete inactivation of *APC*, as documented in previous studies (Miyaki et al, 1993; Sen-Gupta et al, 1993; Palmirotta et al, 1995). On the other hand, truncating *APC* mutations were observed in only 2 of the 16 sporadic cases (12.5%), a proportion significantly lower than in FAP patients ($P = 0.03$, Fisher's exact test), and only in one of these did we detect the inactivation of both alleles.

The fraction of mutated sporadic desmoids observed in this study was lower than that reported by Alman et al (1997*b*), who identified *APC* mutations in three of six cases (50%). However, this difference was not significant ($P = 0.10$, Fisher's exact test), and might be caused by a sampling bias.

It seems unlikely that the low rate of *APC* mutations detected in sporadic cases, in comparison with FAP-related ones, is attributable to a reduced sensitivity of the screening protocol employed, as both groups were analysed using the same approach. However, it cannot be excluded, at least in theory, that sporadic desmoids carry *APC* mutations that lie preferentially in those regions of the gene that were not analysed in this study, including regulatory sequences. It is also possible that in sporadic desmoids the *APC* gene is affected by mutations that are not detectable by the techniques that were used. For example, missense mutations in exonic regions that were analysed only by IVSP would have been missed, as this method only detects nonsense and frameshift mutations. These possibilities, however, would be in contrast with previous observations, which suggest that the development of desmoids mediated by the *APC* gene requires the presence of at least one allele carrying a truncating mutation near or beyond codon 1444 (Palmirotta et al, 1995). In fact, our results are in keeping with this hypothesis, as in all cases, both FAP and sporadic, in which *APC* alterations were identified, at least one allele was mutated, either at germline or somatic level, in a region spanning codons 1450–1558.

Total or partial *APC* gene deletion, a possibility suggested by cytogenetic observations (Bridge et al, 1992, 1996), should also be considered. Unfortunately, this could not be adequately examined in this study, as the search for allele losses affecting *APC* could be performed only in four of the sporadic tumours. All were found to be heterozygous for the flanking markers D5S346, but only one case, no. 2324 with somatic mutations in both *APC* alleles, was informative for the two intragenic polymorphisms investigated.

Even considering the above-mentioned technical limitations, our data suggest that mutations in the *APC* gene contribute to the development of only a small fraction of sporadic desmoids. The protein encoded by *APC* takes part in co-ordinated pathways that control cell to cell adhesion and cell migration (Nathke et al. 1996; Barth et al. 1997). These functions are mediated through binding to different cellular proteins, one of which, the β-catenin, appears to be down-regulated by *APC* itself (Munemitsu et al. 1995). It is conceivable that disturbances in these cellular pathways may promote the uncontrolled growth of mesenchymal cells, which gives rise to desmoid formation. In principle, different genetic mechanisms may be responsible for these disturbances. In FAP patients the inactivation of both *APC* alleles is selected for, because of the presence of germline mutations of the gene. In sporadic cases, other genetic alterations may occur with similar probabilities to *APC* mutations. These alterations might affect genes whose products interact directly or indirectly with the APC protein. Among these, an obvious candidate is the β-catenin gene (*CTNNB1*), which was recently reported to be mutated in colon cancer and in melanoma cell lines (Morin et al. 1997; Rubinfeld et al. 1997). However, genes mapped to chromosomes 8 and 20, that have been found to be frequently trisomic in sporadic desmoids (Fletcher et al. 1995; Mertens et al. 1995; Qi et al. 1996), should also be considered.

The identification of inactivating *APC* mutations in desmoids, although limited to a small proportion of cases in sporadic tumours, might have important consequences for the treatment of these neoplasias. At present, the chemotherapy of desmoids is mainly based on the use of non-steroidal anti-inflammatory drugs (NSAIDs) (Clark and Phillips, 1996). Among these, one of the most commonly employed is sulindac. Recently, sulindac was shown to increase the expression of *APC* mRNA in vitro (Schnitzler et al. 1996), and it has been suggested that this effect might explain, at least in part, the growth inhibition properties of the drug. If this were true, one should expect that sulindac has no or little effect in inducing the regression of desmoids that do not express wild-type APC protein. However, no data are at present available to confirm this hypothesis.

Finally, it must be noted that the two sporadic desmoids in which mutations of *APC* were detected were also the only two non FAP-related tumours that occurred in the abdominal wall. Interestingly, 10 out of the 15 FAP-associated desmoids that have been reported to carry somatic *APC* mutations were also localized in the abdominal wall (Miyaki et al. 1993; Sen-Gupta et al. 1993; Palmirotta et al. 1995 and present study). This might suggest that the presence of somatic *APC* mutations in desmoid DNA, rather than being dependent on the germline status of the patients, reflects the anatomical site of tumour appearance. However, somatic mutations of *APC* were also reported in FAP-related desmoids that originated intra-abdominally (Miyaki et al. 1993 and present study), which is by far the most frequent site of occurrence of desmoids in FAP patients (Jones et al. 1986; Gurbuz et al. 1994), but not in the two intra-abdominal sporadic cases (no. 2429 and 2442) analysed in this study.

### ACKNOWLEDGEMENTS

This work was supported in part by funds from the Italian Ministry of Health and Italian Association and Foundation for Cancer Research (AIRC/FIRC). Equipment at University College of London is provided by Quest Cancer Research. The authors thank Maria T Radice for technical assistance, Mr Mario Azzini for preparation of the figures and Mrs Anna Grassi and Miss Cristina Mazzadi for secretarial assistance.

### REFERENCES

Alman BA, Pajerski ME, Diaz-Cano S, Corboy K and Wolfe HJ (1997a) Aggressive fibromatosis (desmoid tumor) is a monoclonal disorder. *Diagn Mol Pathol* **6**: 98–101

Alman BA, Li C, Pajerski ME, Diaz-Cano S, Wolfe HJ (1997b) Increased β-catenin protein and somatic APC mutations in sporadic aggressive fibromatoses (desmoid tumors). *Am J Pathol* **151**: 329–334

Barth AI, Pollack AL, Altschuler Y, Mostov KE and Nelson WJ (1997) NH2-terminal deletion of beta-catenin results in stable colocalization of mutant beta-catenin with adenomatous polyposis coli protein and altered MDCK cell adhesion. *J Cell Biol* **136**: 693–706

Bridge JA, Sreekantaiah C, Mouron B, Neff JR, Sandberg AA and Wolman SR (1992) Clonal chromosomal abnormalities in desmoid tumors. Implications for histopathogenesis. *Cancer* **69**: 430–436

Bridge JA, Meloni AM, Neff JR, Deboer J, Pickering D, Dalence C, Jeffrey B and Sandberg AA (1996) Deletion 5q in desmoid tumor and fluorescence in situ hybridization for chromosome 8 and/or 20 copy number. *Cancer Genet Cytogenet* **92**: 150–151

Bussey HJR (1975) *Familial Polyposis coli*. Johns Hopkins University Press: Baltimore, MD

Campbell WJ, Spence RA and Parks TG (1994) Familial adenomatous polyposis. *Br J Surg* **81**: 1722–1733

Clark SK and Phillips KS (1996) Desmoids in familial adenomatous polyposis. *Br J Surg* **83**: 1494–1504

Eccles DM, van der Luijt R, Breukel C, Bullman H, Bunyan D, Fisher A, Barber J, du Boulay C, Primrose J, Burn J and Fodde R (1996) Hereditary desmoid disease due to a frameshift mutation at codon 1924 of the APC gene. *Am J Hum Genet* **59**: 1193–1201

Fletcher JA, Naeem R, Xiao S and Corson JM (1995) Chromosome aberrations in desmoid tumors. Trisomy 8 may be a predictor of recurrence. *Cancer Genet Cytogenet* **79**: 139–143

Fodde R, van der Luijt R, Wijnen J, Tops C, van der Klift H, van Leeuwen-Cornelisse I, Griffioen G, Vasen H and Khan PM (1992) Eight novel inactivating germ line mutations at the APC gene identified by denaturing gradient gel electrophoresis. *Genomics* **13**: 1162–1168

Groden J, Thliveris A, Samowitz WS, Carlson M, Gilbert L, Albertsen H, Joslyn G, Stecens J, Spirio L, Robertson M, Sargeant L, Krapcho K, Wolff E, Burt R, Hughes JP, Warrington J, McPherson J, Wasmuth J, Le Paslier D, Abderrahim H, Cohen D, Leppert M and White R (1991) Identification and characterization of the familial adenomatous polyposis coli gene. *Cell* **66**: 589–600

Gurbuz AK, Giardiello FM, Petersen GM, Krush AJ, Offerhaus GJ, Booker SV, Kerr MC and Hamilton SR (1994) Desmoid tumours in familial adenomatous polyposis. *Gut* **35**: 377–381

Jones IT, Jagelman DG, Fazio VW, Lavery IC, Weakley FL and McGannon E (1986) Desmoid tumours in familial polyposis coli. *Ann Surg* **204**: 94–97

Joslyn G, Carlson M, Thliveris A, Albertsen H, Gelbert L, Samowitz W, Groden J, Stevens J, Spirio L, Robertson M, Sargeant L, Krapcho K, Wolff E, Burt R, Hughes JP, Warrington J, McPherson J, Wasmuth J, Le Paslier D, Abderrahim H, Cohen D, Leppert M and White R (1991) Identification of deletion mutations and three new genes at the familial polyposis locus. *Cell* **66**: 601–613

Kinzler KW, Nilbert MC, Vogelstein B, Bryan TM, Levy DB, Smith KJ, Preisinger AC, Hedge P and McKechnie D (1991) Identification of FAP locus genes from chromosomes 5q21. *Science* **253**: 661–664

Li M, Cordon-Cardo C, Gerald WL and Rosai J (1996) Desmoid fibromatosis is a clonal process. *Hum Pathol* **27**: 939–943

Lucas DR, Shroyer KR, McCarthy PJ, Markham NE, Fujita M and Enomoto TE (1997) Desmoid tumor is a clonal cellular proliferation: PCR amplification of HUMARA for analysis of patterns of X-chromosome inactivation. *Am J Surg Pathol* **21**: 306–311

Mackenzie DH (1972) The fibromatoses: a clinicopathological concept. *Br Med J* **4**: 277–281

Mertens F, Willen H, Rydholm A, Brosjo O, Carlen B, Mitelman F, Mandahl N (1995) Trisomy 20 is a primary chromosome aberration in desmoid tumors. *Int J Cancer* **63**: 527–529

Miyaki M, Konishi M, Kikuchi-Yanoshita R, Enomoto M, Tanaka K, Takahashi H, Muraoka M, Mori T, Konishi F and Iwama T (1993) Coexistence of somatic and germ-line mutations of APC gene in desmoid tumors from patients with familial adenomatous polyposis. *Cancer Res* **53**: 5079–5082

Miyaki M, Konishi M, Kikuchi-Yanoshita R, Enomoto M, Igari T, Tanaka, K, Muraoka M, Takahashi H, Amada Y, Fukayama M, Maeda Y, Iwama T, Mishima Y, Mori T and Koike M, (1994) Characteristics of somatic mutation of the adenomatous polyposis coli gene in colorectal tumors. *Cancer Res* **54**: 3011–3020

© Cancer Research Campaign 1998

Miyaki M. Tanaka K. Kikuchi-Yanoshita R. Muraoka M and Konishi M (1995) Familial polyposis: recent advances. *Crit Rev Oncol Hematol* **19**: 1–31

Miyoshi Y. Nagase H. Ando H. Horii A. Ichii S. Nakatsuru S. Aoki T. Miki Y. Mori T and Nakamura Y (1992) Somatic mutations of the APC gene in colorectal tumors: mutation cluster region in the APC gene. *Hum Mol Genet* **1**: 229–233

Morin PJ. Sparks AB. Korinek V. Barker N. Clevers H. Vogelstein B and Kinzler KW (1997) Activation of β-catenin-Tcf signaling in colon cancer by mutations in the β-catenin or APC. *Science* **275**: 1787–1790

Munemitsu S. Albert I. Souza B. Rubinfeld B and Polakis P (1995) Regulation of intracellular β-catenin levels by the adenomatous polyposis coli (APC) tumor-suppressor protein. *Proc Natl Acad Sci USA* **92**: 3046–3050

Nathke IS. Adams CL. Polakis P. Sellin JH and Nelson WJ (1996) The adenomatous polyposis coli tumor suppressor protein localizes to plasma membrane sites involved in active cell migration. *J Cell Biol* **134**: 165–179

Nishisho I. Nakamura Y. Miyoshi Y. Miki Y. Ando H and Horii A (1991) Mutations of chromosomes 5q21 genes in FAP and colorectal cancer patients. *Science* **253**: 665–669

Pack GT and Ehrlich G (1944) Neoplasms of the anterior abdominal wall with special consideration of desmoid tumors: experience with 391 cases and collective review of literature. *Int Abstr Surg* **79**: 177–198

Palmirotta R. Curia MC. Esposito DL. Valanzano R. Messerini L. Ficari F. Brandi ML. Tonelli F. Mariani-Costantini R. Battista P and Cama A (1995) Novel mutations and inactivation of both alleles of the APC gene in desmoid tumors. *Hum Mol Genet* **4**: 1979–1981

Pensotti V. Radice P. Presciuttini S. Calistri D. Gazzoli I. Grimalt Perez AP. Mondini P. Buonsanti G. Sala P. Rossetti C. Ranzani GN. Bertario L and Pierotti MA (1997) Mean age of tumor onset in Hereditary Non-Polyposis Colorectal Cancer (HNPCC) families correlates with the presence of mutations in DNA mismatch repair gene. *Genes Chromosom Cancer* **19**: 135–142

Powell SM. Zilz N. Beazer-Barclay Y. Bryan TM. Hamilton SR. Thibodeau SN. Vogelstein B and Kinzler KW (1992) APC mutations occur early during colorectal tumorigenesis. *Nature* **359**: 235–237

Powell SM. Petersen GM. Krush AJ. Booker S. Jen J. Giardiello FM. Hamilton SR. Vogelstein B and Kinzler KW (1993) Molecular diagnosis of familial adenomatous polyposis. *N Engl J Med* **329**: 1982–1987

Qi H. Dal Cin P. Hernandez JM. Garcia JL. Sciot R. Fletcher C. Van Eyken P. De Wever I and Van Den Berghe H (1996) Trisomies 8 and 20 in desmoid tumors. *Cancer Genet Cytogenet* **92**: 147–149

Reitamo JJ. Scheinin TM and Hayry P (1986) The desmoid syndrome. New aspects in the case, pathogenesis and treatment of desmoid tumor. *Am J Surg* **151**: 230–237

Rubinfeld B. Robbins P. El-Gamil M. Albert I. Porfiri E and Polakis P (1997) Stabilization of β-catenin by genetic defects in melanoma cell lines. *Science* **275**: 1790–1792

Schnitzler M. Dwight T and Robinson BG (1996) Sulindac increases the expression of APC mRNA in malignant colonic epithelial cells: an in vitro study. *Gut* **38**: 707–713

Scott RJ. Froggatt NJ. Trembath RC. Evans DG. Hodgson SV and Maher ER (1996) Familial infiltrative fibromatosis (desmoid tumours) (MIM135290) caused by a recurrent 3′ APC gene mutation. *Hum Mol Genet* **5**: 1921–1924

Sen-Gupta S. Van der Luijt RB. Bowles LV. Meera Khan P and Delhanty JD (1993) Somatic mutation of APC gene in desmoid tumour in familial adenomatous polyposis. *Lancet* **342**: 552–553

Spirio L. Nelson L. Joslyn G. Leppert M and White R (1991) A CA repeat 30–70 Kb downstream from the adenomatous polyposis coli (APC) gene. *Nucleic Acids Res* **19**: 6348

© Cancer Research Campaign 1998